# Chorioamnionitis Causes Kidney Inflammation, Podocyte Damage, and Pro-fibrotic Changes in Fetal Lambs

**DOI:** 10.3389/fped.2022.796702

**Published:** 2022-04-04

**Authors:** Lieke A. Hoogenboom, A. Titia Lely, Matthew W. Kemp, Masatoshi Saito, Alan H. Jobe, Tim G. A. M. Wolfs, Michiel F. Schreuder

**Affiliations:** ^1^Department of Pediatrics, Maastricht University Medical Centre, Maastricht, Netherlands; ^2^Department of Pediatric Nephrology, Radboudumc Amalia Children’s Hospital, Nijmegen, Netherlands; ^3^Department of Obstetrics, Wilhelmina Children’s Hospital Birth Center, University Medical Center Utrecht, University of Utrecht, Utrecht, Netherlands; ^4^Centre for Perinatal and Neonatal Medicine, Tohoku University Hospital, Sendai, Japan; ^5^Division of Obstetrics and Gynaecology, The University of Western Australia, Perth, WA, Australia; ^6^Women and Infants Research Foundation, Perth, WA, Australia; ^7^Department of Obstetrics and Gynaecology, Yong Loo Lin School of Medicine, National University of Singapore, Singapore, Singapore; ^8^Division of Neonatology/Pulmonary Biology, The Perinatal Institute, Cincinnati Children’s Hospital Medical Center, University of Cincinnati, Cincinnati, OH, United States; ^9^Department of Pediatrics, School for Oncology and Developmental Biology (GROW), Maastricht University, Maastricht, Netherlands; ^10^Department of Biomedical Engineering (BMT), Maastricht University, Maastricht, Netherlands

**Keywords:** chorioamnionitis, prematurity, ovine model, mesangium, podocyte

## Abstract

**Background:**

Perinatal complications, such as prematurity and intrauterine growth restriction, are associated with increased risk of chronic kidney disease. Although often associated with reduced nephron endowment, there is also evidence of increased susceptibility for sclerotic changes and podocyte alterations. Preterm birth is frequently associated with chorioamnionitis, though studies regarding the effect of chorioamnionitis on the kidney are scarce. In this study, we aim to unravel the consequences of premature birth and/or perinatal inflammation on kidney development using an ovine model.

**Methods:**

In a preterm sheep model, chorioamnionitis was induced by intra-amniotic injection of lipopolysaccharide (LPS) at either 2, 8, or 15 days prior to delivery. Control animals received intra-amniotic injections of sterile saline. All lambs were surgically delivered at 125 days’ gestation (full term is 150 days) and immediately euthanized for necropsy. Kidneys were harvested and processed for staining with myeloperoxidase (MPO), Wilms tumor-1 (WT1) and alpha-smooth muscle actine (aSMA). mRNA expression of tumor necrosis factor alpha (*TNFA*), Interleukin 10 (*IL10*), desmin (*DES*), Platelet derived growth factor beta (*PDGFB*), Platelet derived growth factor receptor beta (PDGFRB), synaptopodin (SYNPO), and transforming growth factor beta (*TGFB*) was measured using quantitative PCR.

**Results:**

Animals with extended (but not acute) LPS exposure had an inflammatory response in the kidney. MPO staining was significantly increased after 8 and 15 days (*p* = 0.003 and *p* = 0.008, respectively). Expression of *TNFA* (*p* = 0.016) and *IL10* (*p* = 0.026) transcripts was increased, peaking on day 8 after LPS exposure. Glomerular aSMA and expression of TGFB was increased on day 8, suggesting pro-fibrotic mesangial activation, however, this was not confirmed with PDFGB or PDGFRB. The number of WT1 positive nuclei in the glomerulus, as well as expression of synaptopodin, decreased, indicating podocyte injury.

**Conclusion:**

We report that, in an ovine model of prematurity, LPS-induced chorioamnionitis leads to inflammation of the immature kidney. In addition, this process was associated with podocyte injury and there are markers to support pro-fibrotic changes to the glomerular mesangium. These data suggest a potential important role for antenatal inflammation in the development of preterm-associated kidney disease, which is frequent.

## Introduction

Perinatal complications, including preterm birth and inflammatory stress, are associated with an increased risk of developing proteinuria, hypertension, and chronic kidney disease (CKD) in later life (1). Gjerde et al. showed that the hazard ratio (HR) of end stage kidney disease (ESKD) increases with prematurity (HR 1.65) and low birth weight (LBW) (HR 1.61) (2). Subjects with both risk factors had a further increased HR of 2.96. This increased risk fits with the hypothesis of a reduced nephron endowment and subsequent hyperfiltration, as described in the Brenner hyperfiltration theory (3).

In addition to the low nephron endowment, there is increasing evidence that the predisposition for kidney disease has a more complex etiology (1). For example, Menendez-Castro et al. published data on early podocyte alterations secondary to intrauterine growth restriction (IUGR) (4). Moreover, Plank et al. described, in an IUGR rat model, that Thy1.1 -induced nephritis has an aggravated course with more mesangial fibrosis in IUGR animal when compared to non-IUGR animals (5). Additionally, Sutherland et al. found a reduced glomerular surface area was associated with prematurity (6). These studies show that perinatal complications not only affect the number of nephrons, but also the glomerulogenesis.

One of the dominant etiological factors involved in preterm birth is infection and inflammation (7). Chorioamnionitis is a clinically heterogenous condition, the incidence of which is inversely correlated with gestational age. Histologic chorioamnionitis has been shown to occur in up to 50% of cases of preterm birth (7). In cohort studies, (subclinical) chorioamnionitis is rarely taken into consideration when assessing perinatal risk factors for CKD. Although evidence of the effects of chorioamnionitis and inflammation on other organs, such as the brain, lung, intestine, spleen, and skin is ubiquitous, research into the effect on the kidneys is scarce (8–12). Galinsky et al. and Ryan et al. focused on chorioamnionitis and its effect on nephron endowment and found that a single dose of intra amniotic (IA) lipopolysaccharide (LPS), from *Escherichia coli* (a sterile means of modeling chorioamnionitis), leads to a reduction in nephron number, whereas chronic chorioamnionitis simulated by continuous LPS infusion does not. Of these two studies, only Galinsky et al. reported on kidney inflammation and observed no difference in kidney leukocyte infiltration (13, 14). Muk et al. recently published data describing the inflammatory response in the kidney to IA LPS injection and were the first to report both an innate and adaptive immune response in the kidney in the first 8 days after IA LPS injection (15). They also found a simultaneous increase in kidney injury markers neutrophil gelatinase-associated lipocalin (NGAL) and kidney injury molecule-1 (KIM1).

However, the mechanisms that link chorioamnionitis with inflammation in the kidney to the development of proteinuria, hypertension and CKD in later life are poorly understood. The aim of our study was to investigate the impact(s) of premature birth and/or perinatal inflammation on kidney development using an ovine model of pregnancy. We hypothesized that injury to podocytes and the glomerular mesangium may play an important role, as these structures are associated with proteinuria and fibrosis, respectively.

## Materials and Methods

### Experimental Design

The animal procedures were performed with approval of the animal ethics committee of the University of Western Australia (Perth, WA, Australia). The animal experiment was performed exclusively in Australia. After harvesting, the kidneys were transported to Netherlands, which is where the specific tissue analysis was performed.

**FIGURE 1:**
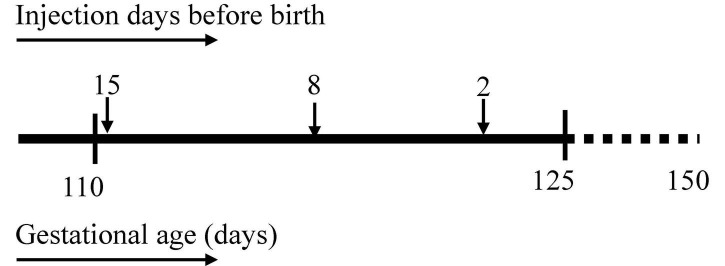
Experimental design. Timeline at the **(top)** represents intra amniotic (IA) lipopolysaccharide injection at 15, 8, and 2 days before sacrifice. Timeline at the **(bottom)** represents gestational age in days with the first IA injection at 110 days, sacrifice at 125 days, and illustrating term date at 150 days gestation.

Twenty-six time-mated ewes with singleton fetuses were randomly allocated into four groups of 5–7 animals per group. An IA injection of 10 mg *E. coli*-derived LPS (O55:B5; Sigma-Aldrich, St Louis, MO, United States) was administered under ultrasound guidance at 2 (*n* = 6), 8 (*n* = 7), or 15 days (*n* = 7) before preterm delivery at 125 days of gestation (term = 150 days) (Figure 1). The control group (*n* = 6) consisted of a combined saline group who received a sham injection with 0.9% saline of the same volume as the LPS injection 2, 8, or 15 days before cesarean section. All fetuses were surgically delivered and immediately euthanized with intravenous pentobarbitone (100 mg/kg). Fetal blood was collected, and the kidneys were snap frozen and stored at –80°C for future analysis.

### Immunohistochemistry

To prepare the tissue for immunohistochemistry, cross sections of the snap-frozen kidney tissue was fixed in formaldehyde 4%, embedded in paraffine and subsequently cut into 4 μm thick sections. All slides were deparaffinized and rehydrated by incubation in xylol and decreasing alcohol concentrations, followed by suppression of endogenous peroxidase activity with H_2_O_2_ and antigen retrieval in citric acid buffer (pH 6.0) for 10 min. The presence of neutrophils was assessed by myeloperoxidase (MPO) staining (1:500, rabbit anti-human, Dako A0398) and detected with a secondary antibody, goat-anti-rabbit-HRP (Jackson 111-035-045). The mesangial response to kidney inflammation was assessed by alpha-Smooth Muscle Actin (aSMA) (1:1000, mouse anti-aSMA, Sigma A2547) and detected with a secondary antibody, polyclonal goat-anti-mouse HRP (Dako p0447). Podocyte damage was assessed with Wilms Tumor-1 (WT1) (1:50) with secondary antibody biotinylated anti-rabbit. Non-specific binding was blocked with 5% normal goat serum (NGS) for the MPO and aSMA staining, and with 100% fetal calf serum (FCS) for the WT1 staining. The primary antibody was visualized with 3-amino-9-ethylcarbazole (AEC) (MPO) or 3,3′-diaminobenzidine (DAB) (aSMA and WT1). All sections were counterstained with hematoxylin.

Myeloperoxidase staining was analyzed by counting the number of MPO positive cells per power field at 200× magnification. After checking MPO was evenly distributed throughout the tissue without clusters of cells, 5 power fields were randomly chosen on each slide using the microscope on 200× magnification and the number of positive cells counted. For the WT1 analysis, 40 glomeruli were randomly selected and WT1 positive cells per glomerulus were manually counted. The number of WT1 positive nuclei were corrected for glomerular surface area to account for possible glomerular hyperfiltration. The slides stained for aSMA were scanned and Leica Qwin Pro v3.4.0 software was used to calculate the percentage aSMA positive area per glomerular surface area of all glomeruli in five randomly chosen power fields.

### mRNA Transcript Expression

Sheep-specific primers were designed for the inflammatory markers tumor necrosis factor-alfa (*TNF*), interleukin-10 (*IL10*), transforming growth factor-beta (*TGFB*) and platelet derived growth factor-b (*PDGFB*), platelet derived growth factor receptor-b (PDGFRB), kidney injury marker desmin (*DES*), and podocyte marker synaptopodin (SYNPO) (Table 1). mRNA was extracted from snap frozen kidney using the Qiagen RNeasy Plus mini kit. The isolated RNA was converted to DNA by reverse transcriptase PCR using the SensiFast cDNA synthesis Kit. Real-time qPCR was performed with the SensiMix SYBR Hi-ROX Kit sing the LightCycles-480 instrument. The primer’s qPCR results were normalized to the geometric mean of the housekeeping genes OVRSP15, GAPDH, and YWHAZ and corrected by Δ*Ct*-values by using the geometric mean of the three housekeeping genes.

**TABLE 1:** Quantitative PCR primers.

Primer	Sequence
TNFA	Forward 5′-GCCGGAATACCTGGACTATGC
	Reverse 5′-CAGGGCGATGATCCCAAAGTAG
IL10	Forward 5′-CATGGGCCTGACATCAAGGA
	Reverse 5′-CGGAGGGTCTTCAGCTTCTC
DES	Forward 5′ TACCAGGACCTGCTCAATGT
	Reverse 5′ GGGCTTGTTTCTCGGAAGTTG
TGFB	Forward 5′-AAAAGAACTGCTGTGTTCGTCA
	Reverse 5′-GACCTTGCTGTACTGTGTGTCC
PDGFB	Forward 5′ CTACCTGCGTCTGGTCAGC
	Reverse 5′ CAGCTCCGTCTTCATCTAAGGAG
PDGFRB	Forward 5′ TGTTGGGCTACTGAATGTGTTG
	Reverse 5′ CAGACAAGCCACAAGTCAACG
SYNPO	Forward 5′ CCAGCTCTGTGAATTGGCTG
	Reverse 5′ GACGCTTGCGTGATTGTTGG

### Statistical Analysis

All statistical analysis ware performed using GraphPad Prism Version 9.0.0 (GraphPad Software, Inc., San Diego, CA, United States). The data was analyzed for normal distribution. Since normality was not reached, comparison between the different experimental groups was performed by using the non-parametric Kruskal–Wallis test. If Kruskal–Wallis indicated a significant difference between groups, the Dunn’s test for multiple comparisons was performed as *post hoc* analysis. *p* < 0.05 was used as a level for significance.

## Results

### Timing of Inflammatory Response in the Kidney

**FIGURE 2:**
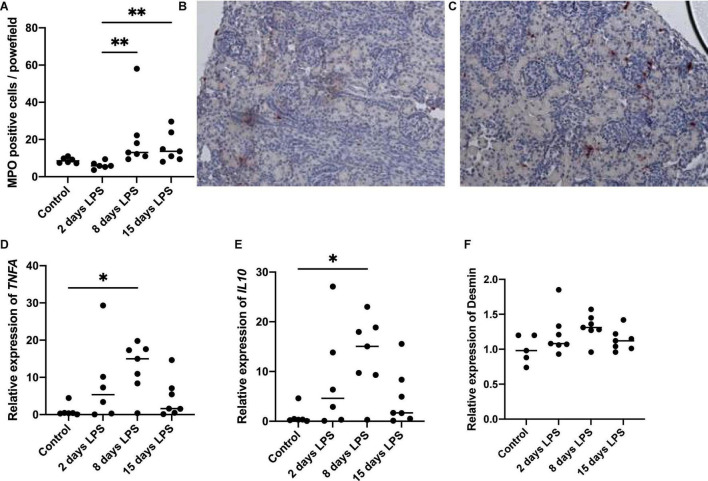
Inflammatory and injury markers in kidney tissue. Each point represents an individual animal, horizontal line marks the median. Data were analyzed using Kruskal–Wallis test and *post hoc* Dunn’s test. **(A)** Average number of myeloperoxidase (MPO) positive nuclei per power field at 200× magnification, ^**^p < 0.01 compared with 2 days lipopolysaccharide (LPS); **(B)** Representative image of MPO staining in control; **(C)** Representative image of MPO staining at 15 days LPS; **(D)** qPCR relative expression of tumor necrosis factor alfa (*TNFA*), *p < 0.05 compared to control; **(E)** qPCR relative expression of *IL10*, *p < 0.05 compared with control; **(F)** qPCR relative expression of desmin.

To assess the presence and timing of inflammation in the kidney, we analyzed the presence of neutrophils and pro-inflammatory cytokines (Figure 2). The number of MPO positive cells started to increase on day 8 after IA LPS injections and remained elevated on day 15. The increase on day 8 and 15 was statistically significant compared to day 2 (*p* = 0.003 and 0.008, respectively). *TNFA* and *IL10* mRNA levels in the kidney started to rise 2 days after IA LPS injection, peaked on day 8 and have returned to baseline on day 15. The peak on day 8 was statistically significant (*p* = 0.016 for *TNFA*, *p* = 0.026 for *IL10*) compared with the controls. The changes observed on days 2 and 15 were not statistically significant for either marker. Desmin qPCR was performed to assess the presence and timing of kidney damage in relation to kidney inflammation, which was non-significantly increased on day 8 (*p* = 0.068).

### Mesangial Response to Chorioamnionitis

**FIGURE 3:**
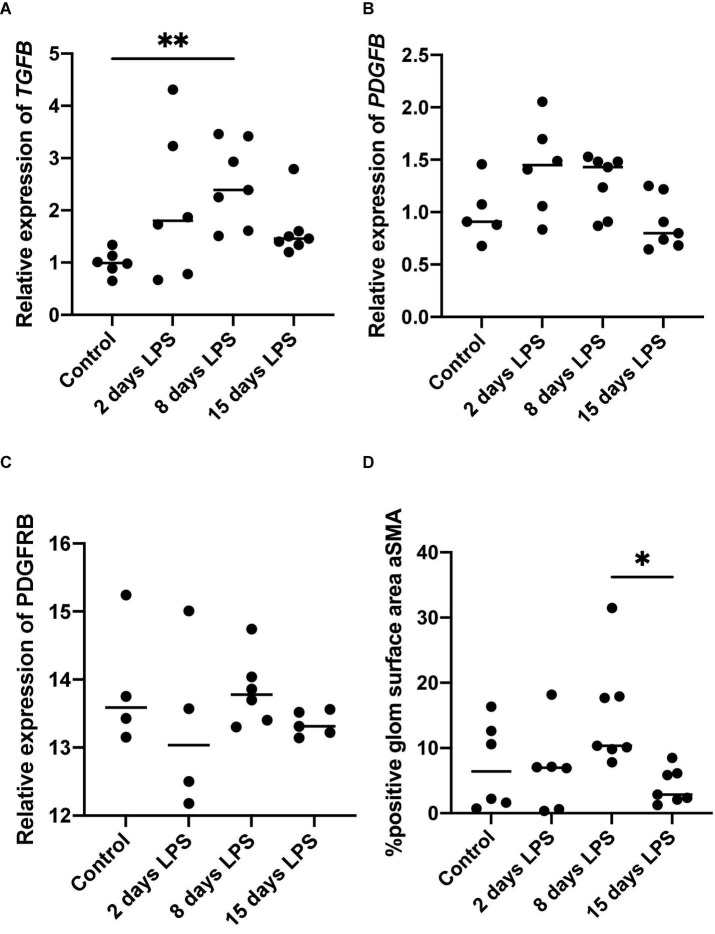
Expression of mesangial pro-fibrotic markers. Each point represents an individual animal, horizontal line marks the median. Data was analyzed using Kruskal–Wallis test and *post hoc* Dunn’s test. **(A)** qPCR relative expression of transforming growth factor beta (*TGFB*), ^**^p < 0.01 compared with control; **(B)** qPCR relative expression of platelet derived growth factor b (*PDGFB*); **(C)** qPCR relative expression of platelet derived growth factor receptor b (PDGFRB); **(D)** Average percentage of glomerular surface area positive for alfa-smooth muscle actin (aSMA) on immunohistochemistry, *p < 0.05 compared with 15 days LPS.

*TGFB*, *PDGFB*, and *PDGFRB* are developmental as well as inflammatory markers. In inflammation, they are known to be early markers associated with pro-fibrotic changes. In our analysis, *TGFB* mRNA expression increased after IA LPS and peaked on day 8 (Figure 3). The peak on day 8 was statistically significant compared to the control group (*p* = 0.004). The increases observed on days 2 and 15 were not statistically significant compared to the control group (*p* = 0.198 and *p* = 0.499, respectively). *PDGFB* and *PDGFRB*, which are stimulated by *TGFB via* the non-SMAD pathway, were not significantly increased after IA LPS injection. *TGFB, PDGFB*, and *PDGFRB* are known to stimulate mesangial cells (16). On immunohistochemistry analysis, the percentage glomerular aSMA positive surface area significantly higher on day 8 compared to day 15. The increase in staining observed on day 8 was not significantly different from that of the control group (*p* = 0.521), or LPS-animals on and day 2 (*p* = 0.193), though appreciating the wide spread in the latter two groups.

### Podocyte Injury

**FIGURE 4:**
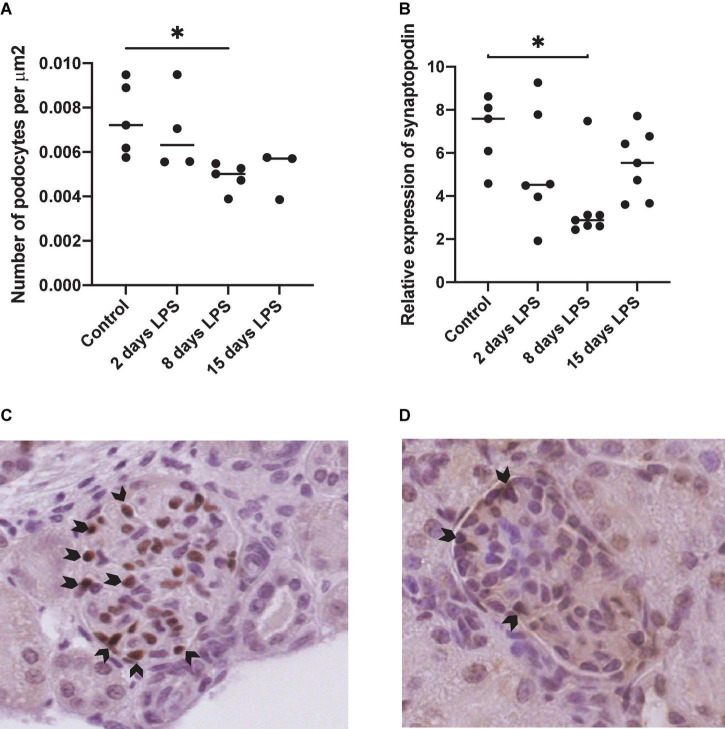
Expression of podocyte markers wilms tumor 1 (WT1) and synaptopodin. Each point represents an individual animal, horizontal line marks the median. Data was analyzed using Kruskal–Wallis test and *post hoc* Dunn’s test. **(A)** Number of WT1 positive nuclei per surface area. *p < 0.05 compared with control; **(B)** aPCR relative expression of synaptopodin. *p < 0.05 compared with control; **(C)** Representative image of WT1 staining in control; **(D)** Representative WT1 staining in 8 days lipopolysacharide (LPS).

Wilms tumor-1 staining was performed as a podocyte-specific marker in the glomerulus. The number of WT1 positive nuclei per glomerular surface was decreased in the days after IA LPS injection with a statistically significant difference on day 8 compared to the control group (*p* = 0.0175) (Figure 4). Synaptopodin mRNA expression, required to maintain the podocyte cytoskeletion, decreased significantly on day 8 after IA LPS injection compared to control animals.

## Discussion

The aim of this study was to gain insight into the consequences of a perinatal inflammatory trigger on the fetal glomerular development, with special focus on the mesangium and podocytes. Our results showed a time-dependent manner of kidney inflammation secondary to chorioamnionitis induced by IA LPS injection in fetal lambs. The kidney inflammatory markers increased between day 2 and 8 post-LPS exposure and peaked on day 8 after IA LPS injection. This ontology coincides with the timing of mesangial activation and podocyte damage, reflected by aSMA increasing and WT1 positive nuclei decreasing on day 8 after IA LPS injection.

Although studies into the mesangial consequences of perinatal inflammation are scarce, one other rat study on antenatal LPS exposure in pregnancy showed similar results (17). The inflammatory response in the kidney promotes tissue repair, however, prolonged activation of pro-inflammatory and pro-fibrotic cell types lead to kidney fibrosis and subsequent kidney function deterioration (16). Increased levels of TNFa have been reported in patients with various kidney diseases, and inhibition with infliximab or etanercept decreases progression of CKD in various animal models (18). TGFB is commonly regarded as having a central role in the development of kidney fibrosis as it drives the extracellular matrix production from mesangial cells (16). Guo et al. showed, in a rat animal model, that antenatal LPS exposure was associated with increased levels of *TNFA*, aSMA, and extracellular matrix deposition in the glomeruli at 20 and 68 weeks of age, thereby confirming the association between chorioamnionitis and fibrotic changes in the glomeruli (17).

The data presented by Guo et al. associate chorioamnionitis with the mesangial fibrosis several weeks after the initial injury. The data presented in our study expand on these findings by directly correlating the timing of the local inflammatory response with the mesangial activation. In the data presented herein, inflammation in the kidney is seen on day 8, with increase in MPO and *IL10*, coinciding with an increase in *TNFA* and aSMA. Surprisingly, aSMA decreases to baseline on day 15 after LPS exposure rather than remaining elevated, as would be expected in prolonged activation of the pro-fibrotic cascade leading to mesangial fibrosis. It can be hypothesized that either mesangial activation following chorioamnionitis has a biphasic pattern, or chorioamnionitis predisposes the mesangium to develop fibrosis on a (mild) second hit, a concept previously demonstrated by Plank et al. in rats after IUGR (5). Further research will be required into the glomerular fibrotic changes in the early period after chorioamnionitis as well as with longer follow-up.

Transforming growth factor beta leads to mesangial activation, proliferation, and extracellular matrix production both directly *via* the TGFB/Smad pathway, and indirectly by stimulating PDGF and PDGFRB, which in turn activate the mesangial cells (18, 19). Our data show an increase in TGFB coinciding with the aSMA upregulation on day 8 after IA LPS injection. However, this same upregulation is not seen in the expression of PDGFB or PDGFRB. It can therefore be argued that the mesangial activation is induced *via* the TGFB/Smad pathway. However, as PDGFB is more often associated with mesangial proliferation, it could also be hypothesized that the increase in PDGFB and PDGFRB is time dependently related to the TGFB peak (Lv et al). Therefore, more research will be necessary to untangle the exact pathway leading to mesangial cell activation after chorioamnionitis and the long-term consequences and identify treatment targets.

Perinatal stress is associated with proteinuria later in life, suggesting perinatal stress impacts podocyte function and/or development. In a rat model, Menendez-Castro et al. found early podocyte alterations associated with IUGR with reductions in nephrin and synaptopodin. Menendez-Castro et al. and Chen et al. both described podocyte damage secondary to IUGR (4, 20). To the best of our knowledge, no research into the effect of chorioamnionitis on podocytes has been published. The sensitivity of WT1 as a marker for podocyte injury has previously been shown by Funk et al., who used multiple models of glomerular injury and found WT1 and desmin to be more sensitive compared to synaptopodin and podocin (21).

In our study, we identified a significant decrease of WT1-positive nuclei on day 8 after LPS injection as well as a decrease in synaptopodin expression. The timing is consistent with the other injury markers used and confirms the hypothesis that chorioamnionitis is associated with to podocyte damage. The reduction of WT1-positive nuclei and synptopodin expression could indicate a reduced formation or a loss of podocytes, in which case the remaining podocytes have to compensate for the lower number by expanding in order to cover the glomerular surface area (22). Recently, a lower number of podocytes in a rat model of IUGR was indeed described (23). On the other hand, the lower number of WT1-positive nuclei can also be explained by podocytes undergoing phenotypic dysregulation, as described by Barisoni et al. in collapsing focal segmental glomerulosclerosis (FSGS) and HIV-associated nephropathy (24). More extensive research will be required to differentiate between a reduced podocyte endowment, podocyte loss or phenotypic dysregulation secondary to chorioamnionitis and to subsequently evaluate the long-term effect on podocytes.

To our knowledge, our study is the first to report histological data associating chorioamnionitis with early kidney injury. We showed that IA LPS injection resulted in an increase in inflammatory markers, such as number of MPO-positive nuclei, and a simultaneous activation of mesangial cells and reduced WT1 positive nuclei. Previously, Muk et al. reported on the kidney response to LPS induced chorioamnionitis in preterm pigs up to day 8 after LPS injection but focused more on the immunological response (15). They showed that prenatal chorioamnionitis induces kidney inflammation *via* activation of both the innate and adaptive immune system. Moreover, they showed that this activation of innate and adaptive immune system, leads to an increase in various kidney injury markers 4 and 8 days after induction of chorioamnionitis. Taken together, these data show that chorioamnionitis leads to a local inflammatory response in the kidney, which, in turn, leads to damage of the glomerular mesangium and podocytes.

A trend toward an increase in desmin qPCR was observed, which may be suggestive of generalized cell damage within the kidney. Zou et al. previously described the time course of desmin expression in relation to glomerular injury. In their study, desmin was unchanged in the first 4 days and increased on day 6 and 10 after induction of glomerular injury (25). This timing of increase is consistent with the trend seen in our data. The absence of a statistical difference in our study could be due to the relative low number of animals in our study, which is an intrinsic limitation of large animal studies. Muk et al. used other markers for generalized cell damage in the kidney, such as caspase 3, and found a significant elevation on day 4 compared to day 9 after LPS injection (15). The difference between our results and those published by Muk et al. could be due to the use of different markers, but can also be attributed to the timing of chorioamnionitis in nephrogenesis. Muk et al. used a pig model in which nephrogenesis continues until 21 days after birth, therefore, chorioamnionitis was induced at 75–80% of nephrogenesis (26). In our study, we used a sheep model in which nephrogenesis continues until approximately 120 days of gestation and nephrogenesis ceases before term birth at approximately 150 days. We induced chorioamnionitis between 90% (for 15 days LPS) and 100% (for 2 days LPS) of nephrogenesis (27). Therefore, these data could indicate that, with progression of nephrogenesis, the injury caused by kidney inflammation secondary to chorioamnionitis on glomerulogenesis decreases.

The timing of glomerular damage in relation to induction of kidney inflammation seen in this study is consistent with that seen in previous studies of nephritis in developing kidneys (15), fully developed kidneys (28, 29) and chorioamnionitis induced inflammation in other organs (10). Ichimura et al. induced mesangioproliferative nephritis by intravenous injection of anti-Thy1.1 antibody in 6-week-old rats (28). PCNA and aSMA, as markers of mesangial proliferation, were not increased on day 3 after injection, peaked on day 5, and slowly decreased on days 8, 14, and 30 after injection. Rampino et al. found, in a similar model in adult rats, that macrophage infiltration was increased on day 7 and 10, but no longer on day 14 after injection of anti-Thy 1.1 antibody (29). Moreover, profibrotic marker PDGFB, and mesangial proliferative markers aSMA and PCNA, were increased 7, 10, and 14 days after injection. These two studies confirm that kidney inflammation and subsequent mesangial activation follows approximately 5–7 days after the initial trigger.

The strengths of this study are twofold. First, the multiple groups delivered at different timepoints after the induction of chorioamnionitis not only allowed us to correlate mesangial and podocyte damage to chorioamnionitis, but it also allowed us to study these changes in a time-dependent manner. Secondly, the applied sheep model accurately resembles human nephrogenesis (27), therefore, accommodating translation to the human situation. A significant limitation of this study is related to the number of animals included in each experimental group, which adversely affects the statistical power. This is a known limitation frequently encountered in large animal research and, therefore, we have reported not only on statistical significance but also trends observed.

In conclusion, we have shown that induction of chorioamnionitis with IA LPS in sheep not only leads to kidney inflammation, but also mesangial activation and podocyte damage. The timing of local inflammation with mesangial activation and podocyte damage is similar to patterns previously described in other nephritis models, however, this is the first study showing these findings in a model of chorioamnionitis. Further research will be required to understand the short- and long-term consequences and relation to observed development of proteinuria and CKD.

## Data Availability Statement

The datasets presented in this study can be found in online repositories. The names of the repository/repositories and accession number(s) can be found in the article/supplementary material.

## Ethics Statement

The animal study was reviewed and approved by Animal Ethics Committee, University of Western Australia, Perth, WA, Australia.

## Author Contributions

LH performed the investigations and wrote the manuscript under supervision of MFS, TW, and AL. MK, MS, and AJ had a significant role in the execution of the animal study. All authors contributed to the article and approved the submitted version.

## Conflict of Interest

The authors declare that the research was conducted in the absence of any commercial or financial relationships that could be construed as a potential conflict of interest.

## Publisher’s Note

All claims expressed in this article are solely those of the authors and do not necessarily represent those of their affiliated organizations, or those of the publisher, the editors and the reviewers. Any product that may be evaluated in this article, or claim that may be made by its manufacturer, is not guaranteed or endorsed by the publisher.
